# Algal Inhibiting Effects of Salicylic Acid Sustained-Release Microspheres on Algae in Different Growth Cycles

**DOI:** 10.3390/ijerph19106320

**Published:** 2022-05-23

**Authors:** Ziqi Fan, Yingjun Wang, Chao Chen, Junrong Li, Yan He, Hong Xiao

**Affiliations:** College of Environmental Sciences, Sichuan Agricultural University, Chengdu 611130, China; fanziqichuannong@163.com (Z.F.); chaochenhc@163.com (C.C.); leelijunrong@163.com (J.L.); heyan@sicau.edu.cn (Y.H.); xiaohong@sicau.edu.cn (H.X.)

**Keywords:** microcystins, chitosan, microspheres, *Microcystis aeruginosa*, microcystin-LR

## Abstract

Microcystis blooms and microcystins caused by eutrophication are harmful to the environment. At present, algicide based on allelochemicals is widely used in algae control. Environment-friendly sustained-release salicylate chitosan microspheres (SA-CS) were prepared by acylation of chitosan and glutaraldehyde. SA-CS was characterized by scanning electron microscopy, Fourier transform infrared spectral analysis, and laser particle sizer. The inhibitory effects of SA-CS on Microcystis aeruginosa at different stages, and the environmental impact of the inorganic index, were studied. The results showed that the mean size of SA-CS was 53.3 μm, the encapsulation rate was 40.66%, and SA-CS had a good sustained-release effect (stable release within 25 days). On the seventh day, a 90% inhibition rate in the lag phase required 105 mg/L of SA-CS, whereas a 90% inhibition rate in the log phase required 675 mg/L of SA-CS. The sensitivity of Microcystis aeruginosa at the lag phase to salicylic acid was about 1.4 times that of the log phase, thus, it is recommended to control the algae in the lag phase. The long-term inhibition effect of SA-CS on algae was detected after adding sufficient SA-CS. In terms of salicylic acid, pH, and dissolved oxygen, no lousy effect was observed for the addition of SA-CS. SA-CS could effectively reduce the concentration of microcystin-LR by 50%. SA-CS is an environment-friendly sustained-release microsphere with good algal inhibition performance for Microcystis aeruginosa.

## 1. Introduction

With the rapid development of aquaculture and food industries, the wastewater generated by these industries will discharge a large amount of nitrogen and phosphorus into fresh water if not properly treated, resulting in an evident trend of eutrophication. The algal blooms, aroused with eutrophication, release microcystins (MCs) and are harmful to the aquatic environment [1]. Microcystins pose a threat to humans through bioaccumulation [2]. According to the monitoring bulletin of 2019, the eutrophication in the Three Gorges reservoir area in China was still severe, and it is the same with some tributaries [3], which had a bad impact on aquatic ecology. Lake Idkul and other lakes in Egypt also show an apparent trend of eutrophication, which negatively impacts the national economy and poses a major threat to the health of local communities [4]. Therefore, how to effectively prohibit algae and microcystins from water has become a problem to be solved. Due to the difference in variable L-amino acids X and Y, microcystin-LR (MC-LR) is the most toxic among MCs [5].

Methods to control blooms include (1) although physical methods, such as flotation, precipitation, and filtration can effectively remove algae, microcystins cannot be removed. Physical algae removal easily causes damage to algae cells and releases intracellular algal toxins, resulting in secondary pollution [6]. (2) Biological methods using algae-eating organisms can improve water quality without causing secondary pollution and are relatively cheap [7]. The disadvantages of biological methods are that the repair time is long, and the effect of algae removal is easily affected by many factors. Yu Ran et al. pretreated algae-containing water with microorganisms, and about 85% of the algae cells and total algal toxins in the pretreated water were effectively removed [8]. (3) Chemical methods, such as electrochemical, chemical reagent oxidation, and allelochemicals inhibition. The electrochemical method is environmentally friendly and has high processing efficiency but a high operating cost. Copper sulfate [9], potassium permanganate, and other chemical substances in the oxidation method can effectively remove algae while killing algal, resulting in the release of microcystins. Allelochemicals have specific toxicity to algae and can degrade microcystins.

In recent years, allelochemicals have been considered safe and have been widely studied. When barley straw is placed into algae fluid, allelopathic substances in straw can inhibit algal growth, and straw, as an adsorbent, can absorb phosphorus and prevent algal growth [10]. This approach is successful in most cases and has no additional effects. Allelochemicals are carriers of allelopathy, including salicylic acid (SA), gallic acid, and catechol, found in many higher plants [11]. SA, as an allelochemical, has strong allelopathic inhibition at low concentrations [12]. Many studies have successfully isolated SA and confirmed its allelopathic inhibitory effect on algae [13,14]. Kovacik et al. [15] showed that SA could effectively inhibit a variety of algae; Wang et al. [16] proved that a variety of phenolic acids could inhibit the growth of Microcystis aeruginosa. However, if SA is added directly to the water, SA at high concentrations is difficult to diffuse and easily raises ecological problems. Because SA is easy to photolysis, direct addition of SA cannot inhibit algae growth for a long time. The problems mentioned above can be avoided by developing SA products with slow-release functions.

As a natural raw material, chitosan (CS) contains a large amount of hydroxyl and amine [17], which has a certain inhibitory effect on algae [18]. Meanwhile, as an adsorption material, CS can also absorb various acids, phenols, and heavy metals [19,20] but with a defect of low mechanical strength [21]. Therefore, many scholars have improved the strength of CS. The carbonization of CS can improve stability and adsorption capacity [22]; the hydrothermal method is used to make CS magnetic [23]; the emulsification crosslinking method is used to make CS have a better sustained-release effect [24]. The experimental temperature of the emulsified crosslinking method was in the normal range, which was beneficial to maintaining drug activity and stability. Meanwhile, the particle size of the microspheres prepared by the emulsified crosslinking method could be controlled at the nanometer level.

It is generally accepted that algal sensitivity increases with a decrease in algal density [25]. The sensitivity of Microcystis aeruginosa to Ethyl 2-methylacetoacetate and barley straw in the lag phase was 3.3 and 1.4 times higher than that in the log stage [26]. However, the sensitivity of grass plants to Pseudomonas aeruginosa decreased with the increase in algal density [27]. Therefore, it is necessary to determine a more appropriate stage through experiments to reduce the dosage and impact on the environment.

Therefore, in this study, the emulsification crosslinking method prepared salicylic acid chitosan microspheres (SA-CS). The sustained-release protective effects of microspheres on SA, long-term algicidal ability, and environmental effects of microspheres on Microcystis aeruginosa were investigated. This study provides a method for controlling algal blooms effectively under eutrophication with low ecological risk.

## 2. Materials and Methods

### 2.1. Experimental Materials

*Microcystis aeruginosa* (FACHB-930) was purchased from the Freshwater Algae Culture Collection at the Institute of Hydrobiology. CS, with a deacetylation degree of >80%, and analytical pure SA were purchased from Chengdu Kelong Chemical Co., Ltd., Chengdu, China.

### 2.2. Algae Culture Conditions

We strictly met the requirements of the aseptic technique and transferred algae fluid into a sterilized small flask (50–200 mL) from test tubes on the clean bench. The volume ratio of algae solution and BG-11 medium was 1:3. Then, algae were cultivated in the light incubator with a temperature of 25 ± 0.5 °C and a light intensity of 2000–2100 Lx, with 12 h light and 12 h dark operation.

### 2.3. Preparation of SA-CS

CS (350 mg) was dissolved in 30 mL of 1% acetic acid solution, then 60 mg of SA was added in anhydrous ethanol (2 mL) and stirred for 0.5 h. Then, the mixture was added to the mixed oil phase (90 mL of liquid paraffin, 6 mL of Span80) drop-by-drop within 10 min, and the mixture was emulsified into water/oil emulsion using the agitator. Then, 8 mL of 25% glutaraldehyde was added drop-by-drop, and the crosslinking reaction occurred at 50 °C for 8 h. The products were separated by centrifugation, then washed with ethanol and petroleum ether three times, respectively. By vacuum drying the filtered residue SA-CS was obtained.

### 2.4. Experimental Methods

#### 2.4.1. Release Properties of SA-CS

An amount of 80 mg SA-CS and about 2.7 mg of SA were added to the 150 mL conical flask separately and 100 mL of BG-11 medium was added. The sample was placed separately for 25 days at 25 °C in light and darkness. The SA concentration was determined by timing sampling. Sampling time was 1, 2, 3 days, and then every three days.

#### 2.4.2. Characterization of SA-CS

The dried SA-CS was characterized by scanning electron microscopy (SEM), a laser particle size analyzer and FT-IR. FT-IR spectroscopy (Spectrum two, PerkinElmer, Waltham, MA, USA) was performed at 4000~400 cm^−1^ using the potassium bromide disc technique to measure the absorbance of pure CS and SA-CS. The surface morphology of SA-CS was examined using a Gemini SEM300 (Carl Zeiss AG, Oberkochen, Germany) by adjusting the appropriate magnification.

#### 2.4.3. Inhibitory Effect of SA-CS on Algae and Its Environmental Effect on Algal Fluid during Lag and Log Phases

An amount of 3, 9, 15, 21, and 27 mg of SA-CS were added into 200 mL of lag phase algal liquid (algal cell density was about 1.54 × 10^6^ cell/mL, chlorophyll *a* (Chl-*a*) concentration was about 180.5 μg/L), and measured once a day for seven days. Amounts of 35, 60, 85, 110, and 135 mg SA-CS were added into 200 mL of log phase algal liquid (algal cell density was about 1.0 × 10^7^ cell/mL, Chl-*a* concentration was about 980 μg/L) and measured once a day for seven days. The effect of the SA-CS dosage on the algal-killing effect at different phases was observed. The algal liquid was taken every day to measure pH, the concentration of SA, the concentration of dissolved oxygen (DO), and the concentration of Chl-*a*. The MC-LR was sampled on days 1, 3, 5, and 7 to observe the influence of the SA-CS algal suppressant on the water environment.

### 2.5. Method for the Determination of Experimental Indexes

#### 2.5.1. Determination of Chl-*a*

Extraction and determination of Chl-*a* according to “Water and wastewater monitoring and analysis methods” (fourth edition) operation [28], occurred with some improvement. An amount of 2 mL of algal liquid was taken and ultracentrifuged in a TGL-16B ultracentrifuge (Shanghai Anting Scientific, Shanghai, China) at 10,000 r/min for 5 min. After centrifugation, the liquid part was abandoned, and 2 mL of 90% acetone (volume ratio) was added to the shake well and then extracted at 4 °C for 24 h. After extraction, the extract was centrifuged again at 10,000 r/min for 5 min. The absorbance of the supernatant was measured on a UV-3100 UV-visible spectrophotometer (Mapada Instruments, Shanghai, China) at 750, 663, 645, and 630 nm with 90% acetone as a reference. The calculation formula of Chl-*a* is as follows:

C_Chl-*a*_ (mg/L) = 11.64(OD_663_ − OD_750_) − 2.16(OD_645_ − D_750_) + 0.1(OD_630_ − OD_750_) (1)

where 11.64, 2.16, and 0.1 are the specific absorption coefficients of 90% acetone solution at 663, 645, and 630 nm, respectively.

#### 2.5.2. Methods for Sampling and Determination of SA

The extraction and concentration of SA were determined by the ferric chloride color development method of Qian [29]. Under aseptic conditions, 2 mL of algal liquid was absorbed, centrifuged at 10,000 r/min for 5 min, and the supernatant was transferred to a 10 mL volumetric flask. Then, 0.01 mol/L hydrochloric acid was added, and a drop of ferric chloride solution was added. After shaking, the absorbance was measured at 530 nm with the blank sample as a reference.

#### 2.5.3. Sampling and Determination Method of MC-LR

The extraction and determination methods of MC-LR were determined by HPLC, according to Zhao et al. [30]. Sample pretreatment ((solid-phase extraction (SPE)): (1) Filtration: Absorb 12 mL algal liquid and use the water filter of 0.45 μm membrane to collect the filtrate. (2) Activation: SPE column was activated with 6 mL of methanol and 6 mL of high purity water. (3) Enrichment: 10.0 mL of filtrate was added for enrichment after activation of the SPE column. (4) Elution: After enrichment, impurities were removed with 6 mL of high purity water and 6 mL of 10% methanol, and then MC-LR was washed twice with 80% methanol (3 mL each time) to elute MC-LR. The eluent was concentrated to less than 1 mL with a rotary evaporator, and the volume was fixed to 1 mL with high purity water. The samples were obtained through the above operations.

MC-LR was determined with the Agilent 1200 liquid chromatography system (Agilent Technologies Inc., Palo Alto, CA, USA) Chromatographic conditions: Chromatographic column: C18 reversed-phase column with a column length of 250 mm, inner diameter of 4.6 mm and filler particle size of 5 μm; chromatographic column temperature: 40 °C; mobile phase: high purity water (containing 0.05% trifluoroacetic acid): methanol = 65:35; flow rate: 1.0 mL/min; detector: UV detector, wavelength 238 nm.

#### 2.5.4. Determination of DO and pH

The pH was measured by a phS-3C pH meter (Chengdu Ark Technology Development Company, Chengdu, China). The DO concentration was measured by a HQ30D dissolved oxygen meter (Hach Company, Loveland, CO, USA).

### 2.6. Data Processing

All the above tests were set in parallel three times, and the test results were the average of the three tests. All data in this study were calculated by Excel 2010. SPSS 17.0 was used to conduct a one-way ANOVA on the data. Duncan’s method was used to conduct multiple comparisons. Different letters or a *p* < 0.05 indicated significant differences between the results. Origin 2017 was used to draw the data graph, in which the error bar represents the standard deviation between the three tests.

## 3. Results and Discussion

### 3.1. SA Release Characteristics of SA-CS

**Figure 1:**
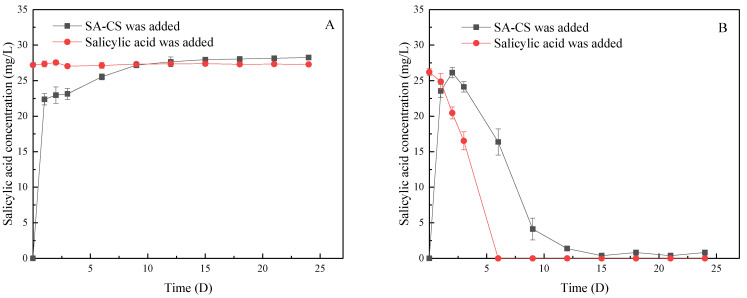
Influence of the light and dark conditions on SA in water: (**A**) shows the effect of darkness on SA and SA-CS; (**B**) shows the effect of light on SA released from SA and SA-CS.

Since SA in the solution would be photolyzed, and there was almost no photodecomposition under darkness [31], SA-CS was placed in a dark environment for desorption to study the actual release of SA by SA-CS. The desorption of SA-CS is shown in Figure 1A, in which the concentration of SA continued to increase, but the speed decreased. Within 25 days, the SA concentration kept increasing, indicating that SA-CS had an excellent sustained-release effect. The release of SA reached 80.07% in the first 24 h, and the effective encapsulation rate of SA-CS was 41.19% after 15 d. The kinetic release data satisfy the first-order and second-order kinetic equations as follows:

First-order dynamic equation: Qeq = 4.10 × (1 − exp(−25.02 × t)) R^2^ = 0.944 (2)

Second-order dynamic equation: Qeq = 183.40 × t/(1 + 7.01 × t) R^2^ = 0.978 (3)

In order to explore the protective effect of SA-CS on SA, this study put quantitative SA-CS and SA microspheres with the same release amount into a culture medium for aeration cultivation under the light. It can be seen from Figure 1B that SA experienced gradual photolysis within 6 days until it cannot be checked, and the residence time is short. SA released from SA-CS could effectively maintain relatively high concentrations (4 mg/L) within 9 days. These results indicated that SA-CS could effectively protect the biological activity of SA and prolong the action time of SA. After 9 days, the concentration of SA in the medium remained near 0.3 mg/L, indicating that SA-CS was still releasing SA with good, sustained release.

### 3.2. Composition and Microstructure of CS and SA-CS

**Figure 2:**
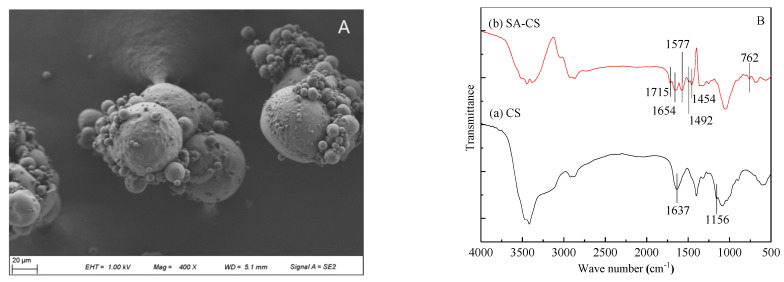
SEM of SA-CS and IR spectra of CS and SA-CS: (**A**) SEM of SA-CS; (**B**) IR spectra of (a) CS and (b) SA-CS.

According to the data measured by the laser granulometer and Figure 2A, the average diameter of SA-CS microspheres is 53.3 μm. Changes in parameters, such as the low oil-water ratio and low acetic acid concentration increase the particle size of the microspheres and microsphere agglomeration probability during molding [32].

CS usually contains amino and hydroxyl groups, while SA has a benzene ring structure, carboxyl, and hydroxyl groups. From Figure 2B, it can be found that CS has characteristic peaks of 1637 cm^−1^ (N–H deformation vibration) and 1156 cm^−1^ (C–O–C tensile vibration) [24]. Compared with CS, SA-CS showed a tensile vibration peak of C=N at 1654 cm^−1^, indicating that CS and glutaraldehyde successfully crosslinked and formed the Schiff base. In the spectrum of SA-CS, the benzene ring C=O peak appeared at 1715 cm^−1^, the stretching vibration peak of the benzene ring appeared at 1577, 1492, and 1454 cm^−1^ [33], and the benzene ring C–H bond characteristic peak appeared at 762 cm^−1^. The characteristic peaks of SA indicate that SA-CS successfully loaded SA.

### 3.3. Inhibitory Effect of SA-CS on Algae during Lag Phase and Log Phase

**Figure 3:**
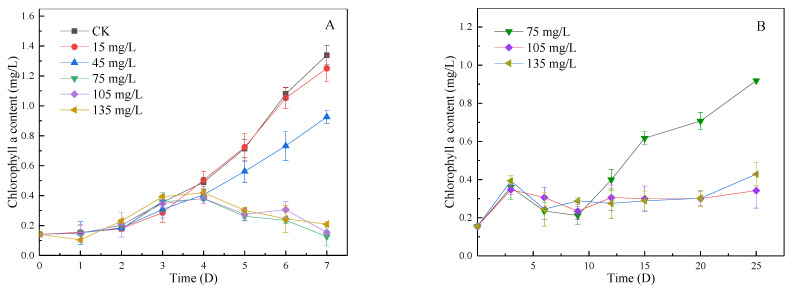

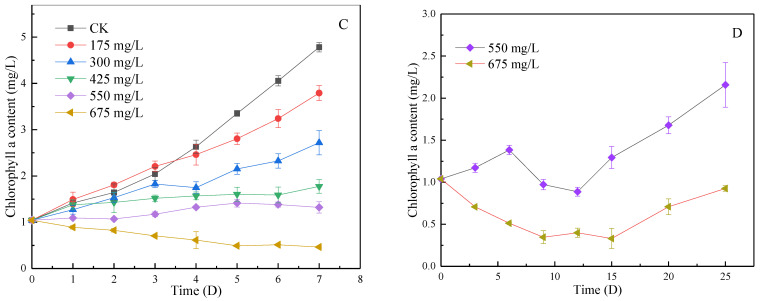
Short-term and long-term Chl-*a* concentration change of lag phase and log phase: (**A**) short-term Chl-*a* concentration change of lag phase; (**B**) long-term Chl-*a* concentration changes of lag phase; (**C**) short-term Chl-*a* concentration change of log phase; (**D**) long-term Chl-*a* concentration changes of log phase.

As shown in Figure 3A, when algae were in the lag phase, there was no significant difference in Chl-*a* concentration among groups in the first three days, indicating that the inhibition effect of microspheres on algae was weak in the initial stage. From the fourth day, Chl-*a* concentration in all groups except the 15 mg/L group was significantly lower than that in the blank group, indicating that SA-CS began to inhibit algae growth. The inhibitory effect of SA-CS on algae was proportional to the dose of SA-CS. When the dosage was more than 75 mg/L, the magnitude of inhibition reached the maximum, and the inhibition rate was close to 90% on the seventh day. According to the effect of algae control, the 75, 105, and 135 mg/L groups were selected for long-term observation. As shown in Figure 3B, there was no difference between the three groups in the first 12 days, and a 90% algae-killing rate was achieved and maintained on the ninth day. After 12 days, algae in the 75 mg/L group regained growth, while the remaining two groups could be inhibited on algae for at least 20 days. Therefore, in the lag phase, when the SA-CS dose reaches 105 mg/L, algae growth can be continuously inhabited for at least 20 d.

As shown in Figure 3C, when *Microcystis aeruginosa* was in the log phase, the growth of algae was inhibited in all experimental groups. When the dose was 175–425 mg/L, SA-CS could only inhibit the growth of microcystis. The higher the dose, the stronger the inhibition; in the group of 675 mg/L, the biomass of *Microcystis* decreased and remained stable on day 4. The algal elimination rate could reach 90% after 7 d in the group of 675 mg/L. According to Figure 3C results, the 550 and 675 mg/L groups were selected for long-term observation. When the dosage reached 675 mg/L, SA-CS had a significant inhibitory effect on algae, and the algal-killing rate reached 90% after 7 days. Algae recovered in 15–20 days, indicating that SA-CS could keep a high efficiency for a long time under 675 mg/L (Figure 3D). The ratio of the SA-CS dosage to algae concentration in the log phase was more than 40% of that in the lag phase. The lag phase had a longer algal inhibition time, indicating that SA-CS is more suitable for algal inhibition in the lag phase. Therefore, in real-life applications, when water bloom occurs in the eutrophic water body, adding SA-CS can achieve a better algae inhibition effect. The longer the bloom exists, the worse the inhibitory effect of SA-CS on algae.

### 3.4. Effects of SA-CS on Environmental Factors in Algal Fluid during Lag and Log Phase

#### 3.4.1. Change in SA Concentration in Algal Fluid during Lag Phase and Log Phase

**Figure 4:**
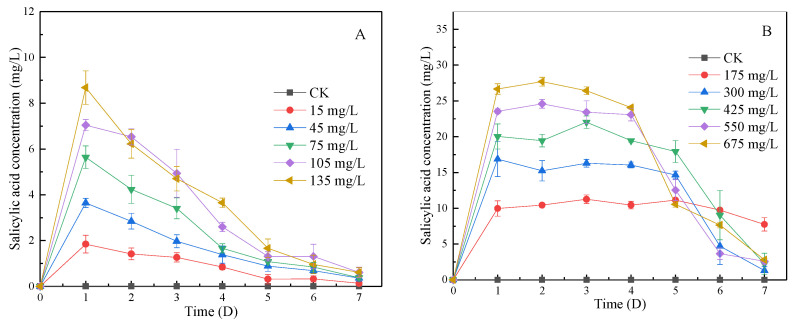
The effect of SA-CS on the concentration of SA in algal liquid at lag phase and log phase: (**A**) the effect of SA-CS on SA at lag phase; (**B**) the effect of SA-CS on SA at log phase.

SA can inhibit or even kill algae by destroying cell membranes. As shown in Figure 4A, in the lag phase, the concentration of SA increased significantly in each group, and the concentration was positively proportional to the dosage of SA-CS (R^2^ = 0.996). After the first day, the SA concentration gradually decreased to near zero within 6 days due to the photolysis of SA. Under the light, SA is easily photolyzed and produces malonic acid and maleic acid [34]. Malonic acid has been shown to have allelopathic algal inhibition [35]. Combined with Figure 3, when the concentration of SA was the highest on the first day, the algae was not inhibited. However, they began to be inhibited on the fourth day, indicating that the reason for the algae inhibition was due to the combined effect of SA and its photolysis products. In the log phase, Figure 4B shows that SA in all experimental groups showed a trend that increased firstly, was kept stable in the middle period and finally decreased. The concentration of SA remained stable in the medium term, which was the high concentration and opacity of algal liquid in the log phase, which weakens the photolysis of SA [30], making the photolysis rate close to the rate of release from SA-CS. The higher dosage of SA-CS could improve the inhibition effect on algae, speed up the improvement of transparency of algal liquid, and increase the decrease rate of SA in the later period. The 48 h semi-lethal concentration of SA by *Moina macrocopa* was >100 mg/L [36]. In contrast, the effective SA maximum concentration that can inhibit algae growth is less than 30 mg/L, indicating that SA released by SA-CS has little impact on the environment.

#### 3.4.2. Change of pH in Algal Fluid during Lag Phase and Log Phase

**Figure 5:**
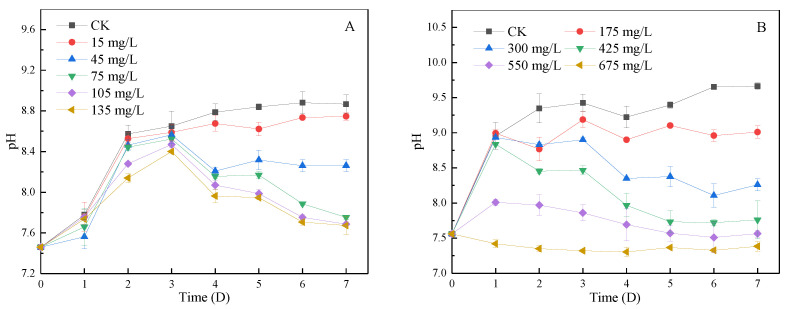
The effect of SA-CS on the concentration of pH in algal liquid at lag phase and log phase: (**A**) the effect of SA-CS on pH at lag phase; (**B**) the effect of SA-CS on pH at log phase.

The pH will affect the growth and reproduction of various organisms in water, and the growth of algae will also lead to pH changes [37,38]. As shown in Figure 5A, the pH of each group in the lag phase increased in the first three days without significant difference, indicating that the inhibition of SA-CS on early algae was weak and algae growth was good. From day 4, the pH of all groups showed a significant difference, and the pH of the 45, 75, 105, and 135 mg/L groups began to decrease. After 7 days, the pH of the 75, 105, and 135 mg/L groups was below 7.8, and the water pH returned to normal. As shown in Figure 5B, for the blank group, the growth amount of algae in the log phase was positively correlated with the change in pH, similar to Su et al. [39]. On the first day, the pH of the 550 mg/L group increased by 0.5, the pH of the 675 mg/L group did not change significantly, and the pH of the other groups increased by about 1.4. When the dosage of SA-CS is 550 mg/L or above, the pH of the water can be restored to the normal level. Obviously, with the decrease in the SA-CS dosage, the inhibition’s magnitude decreased, the absorption effect of HCO^3−^ was strengthened, and pH increased faster. Cyanobacteria photosynthesis can make HCO^3−^ enter cells through an anion exchange mechanism, exchange OH^−^, and increase the pH of water [38]. SA can kill algae so that the HCO^3−^ in the cells returns to the water, restoring the original pH of the water.

#### 3.4.3. Change of DO in Algal Fluid during Lag Phase and Log Phase

**Figure 6:**
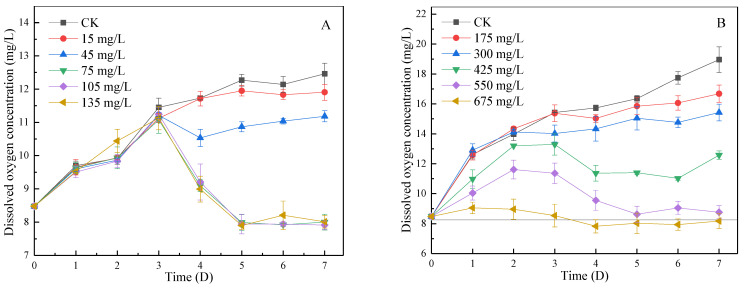
The effect of SA-CS on the concentration of DO in algal liquid at lag phase and log phase: (**A**) the effect of SA-CS on DO at lag phase; (**B**) the effect of SA-CS on DO at log phase.

DO is an essential component of aerobic organisms. Lack of DO will directly lead to the death of aerobic organisms, which will significantly impact the ecological diversity of water [40]. In the lag phase, as shown in Figure 6A, DO in CK and the group with a dosage less than 45 mg/L kept increasing at the initial stage and remained stable, with the maximum concentration of DO increased up to 12.46 mg/L. The trend of DO in the 75, 105, and 135 mg/L groups were consistent with that in CK for the first 3 days, however, the DO concentration decreased rapidly from the fourth day to 7.39 mg/L. Eventually, the concentration of DO remained near the saturated dissolved oxygen concentration at 25 °C. In the log phase, as shown in Figure 6B, in the group with supplemental levels less than 300 mg/L, DO show an overall upward trend on day 7. The concentration of DO in the 475 mg/L and above groups increased on days 1 to 2, decreased on days 3 to 4, and finally also stabilized near the saturated dissolved oxygen concentration at 25 °C (*p* < 0.05).

The rise in DO in the initial stage was caused by the fact that the oxygen production capacity was greater than the oxygen consumption capacity when there was little or no inhibition. The decrease in DO concentration is mainly due to the inhibition of photosynthesis by SA, which reduces the oxygen production of algae [41]. At the same time, the reduction of algae quantity will also lead to the reduction in oxygen production capacity and will also reduce the DO concentration. In neither the log phase nor lag phase, reduction of the DO concentration is limited to the part that exceeds the saturated dissolved oxygen, and the concentration of DO in water remains near the saturated dissolved oxygen concentration, which has little impact on the environment.

#### 3.4.4. Change of MC-LR Concentration in Algal Fluid during Lag Phase and Log Phase

**Figure 7:**
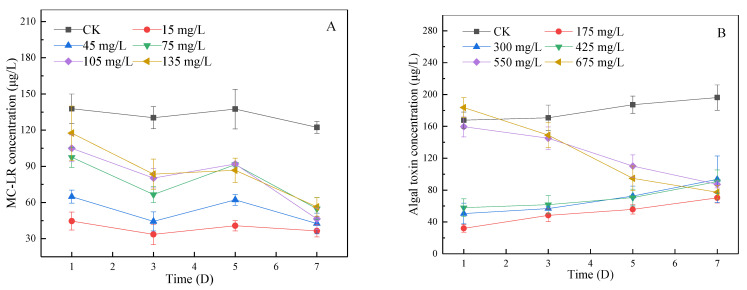
The effect of SA-CS on the concentration of MC-LR in algal liquid at lag phase and log phase: (**A**) the effect of SA-CS on MC-LR at lag phase; (**B**) the effect of SA-CS on MC-LR at log phase.

MC-LR, one of the most studied MCs [42], is highly toxic. The World Health Organization and most countries determine an MC-LR concentration to measure MCs concentration in water. As shown in Figure 7A, the concentration of MC-LR in the blank group was 120–160 μg/L during the delay period, while the concentration in the experimental group was 40–110 μg/L. The addition of SA-CS reduced the concentration of MC-LR in algal liquid. As shown in Figure 7B, the concentration of MC-LR in the blank group increased continuously within 7 days in the log phase, indicating that algae in the log phase will release MCs into water [43]. When the dose of SA-CS was less than 550 mg/L, the concentration of MC-LR continued to increase to about 100 μg/L. Under the low dose condition, the inhibition degree of algae is low, and MCs are released normally. When the dose of SA-CS was greater than 550 mg/L, high doses of SA caused damage to the structure of the algal. Intracellular free radicals were allowed to enter the algal fluid to react with MC-LR [44], thus leading to a decline in the concentration of MC-LR. Comparing experimental groups, the more SA-CS was added, the higher the initial MC-LR concentration was. The increase of MCs is that the dosage of SA-CS increases and enhances the inhibitory effect on algae, resulting in an increase in algal death and the total amount of MC-LR released from algae.

Under similar conditions, *Microcystis* with an initial chlorophyll concentration of 1 mg/L was destroyed by copper ions for 7 days, and the concentration of MCs in water increased by 1.65 times [45]. After adding SA-CS, the concentration of MC-LR decreased by more than 50% in both the log phase and retarded phase, indicating that SA-CS had a good degradation effect on MC-LR.

## 4. Conclusions

SA-CS was prepared from CS and SA with glutaraldehyde as the crosslinking agent to synthesize sustained-release regent to prevent blooms. The mean size of SA-CS was 53.3 μm. SA-CS had good sustained-release performance within 25 days. SA-CS could effectively prohibit the growth of cyanobacteria in the lag and log phase, however, the dosage is different. The sensitivity of *Microcystis aeruginosa* in the lag phase to SA-CS was 1.4 times that of the log phase, and the effective inhibition time of the slow stage was longer, indicating that the material is more suitable for algal inhibition in the lag phase. When added at an adequate dose, SA-CS can inhibit algae growth at least for 15 days, and it is environmentally friendly in terms of SA, MC-LR, pH, and DO in both the lag and log phases.

## Data Availability

Not applicable.
